# Relations of mother’s sense of coherence and childrearing style with child’s social skills in preschoolers

**DOI:** 10.1186/s13034-017-0147-6

**Published:** 2017-03-07

**Authors:** Rikuya Hosokawa, Toshiki Katsura, Miho Shizawa

**Affiliations:** 1 Graduate School of Medicine, Kyoto University, Yoshida-Konoe-cho, Sakyo-ku, Kyoto, 606-8501 Japan; 2 School of Nursing, Nagoya City University, Nagoya, Aichi Japan; 3 3 Graduate School of Nursing, Kyoto Prefectural University of Medicine, Kyoto, Japan

**Keywords:** Sense of coherence, Childrearing, Social development, Social skills, Preschool children

## Abstract

**Background:**

We examined the relationships between mothers’ sense of coherence (SOC) and their child’s social skills development among preschool children, and how this relationship is mediated by mother’s childrearing style.

**Methods:**

Mothers of 1341 Japanese children, aged 4–5 years, completed a self-report questionnaire on their SOC and childrearing style. The children’s teachers evaluated their social skills using the social skills scale (SSS), which comprises three factors: cooperation, self-control, and assertion.

**Results:**

Path analyses revealed that the mother’s childrearing mediated the positive relationship between mother’s SOC and the cooperation, self-control, and assertiveness aspects of children’s social skills. Additionally, there was a significant direct path from mother’s SOC to the self-control component of social skills.

**Conclusions:**

These findings suggest that mother’s SOC may directly as well as indirectly influence children’s social skills development through the mediating effect of childrearing. The results offer preliminary evidence that focusing on support to improve mothers’ SOC may be an efficient and effective strategy for improving children’s social skills development.

## Background

Sense of coherence (SOC), a concept developed by Antonovsky [1], refers to an individual’s personal ability to cope with stressors. Specifically, SOC has been defined as a ‘global orientation expressing the extent to which one has a pervasive, enduring though dynamic feeling of confidence that (i) the stimuli deriving from one’s internal and external environments in the course of living are structured, predictable, and explicable (comprehensibility); (ii) the resources are available to one to meet the demands posed by the stimuli (manageability); and (iii) these demands are challenges, worthy of investment and engagement (meaningfulness)’ [1, 2]. SOC is a theoretical concept that stems from salutogenesis, which focuses on what factors promote health and well-being (as opposed to factors that cause disease, which have been the focus of most models of health) [3–6]. SOC was conceived as a salutary factor (i.e. a health factor) according to the salutogenic model, which states that in order to obtain well-being, it is important for people to focus on their own resources and capacity for coping [7]. The salutogenic model is an important contribution to the theoretical underpinnings of health promotion, which is being advocated by the World Health Organization [8, 9].

SOC influences an individual’s resources for coping with stressful situations. Individuals are always exposed to stress—indeed, it is part of the human environment. Numerous studies on the relationship between SOC and stress have determined that people with a strong SOC tend to cope with stressful situations better, thereby leading to improved well-being and health status. People with high SOC also tend to perceive situations as manageable and meaningful, and view stressors as important challenges worth facing. They tend to be flexible and able to draw on appropriate resources to overcome a situation [10]. In contrast, people with poor SOC tend to be more vulnerable to stress and its negative health effects [5, 11, 12].

SOC appears to be closely associated with health, particularly mental health. SOC is, for instance, inversely related to psychological distress and psychiatric symptomatology (e.g. stress, depression, and anxiety); it appears to mitigate the negative impact of life stress [9–16]. Furthermore, SOC is positively related to psychological well-being [17] and quality of life [18]. On the other hand, poor SOC is related to life stress, psychological distress, and psychiatric symptomatology; it appears to enhance the negative impact of life stress [19]. Depending on levels of SOC, SOC can be either a positive or a negative factor that affects mental health functioning.

SOC also appears to be significantly associated with parenting stress (e.g. stress originating from the parenting role) [20]. Specifically, SOC appears to be positively related to parents’ self-esteem and inversely related to parental stress and depression [21, 22]. Parents are faced with multiple stressors throughout their child’s development, such as decisions on what constitutes effective parenting strategies, managing child behaviour, financial responsibilities, health concerns, and educational responsibilities [23]. Studies suggest that such stressors have a major impact on the child [23, 24]. Especially, maternal stress demonstrated notable negative impacts on children’s outcomes [24]. Maternal stress specifically appears to affect children’s social functioning and strongly predicts their adjustment, including development of internalizing (e.g. anxiety and depression) and externalizing disorders (e.g. inattention, defiance, impulsivity, and aggression) [25–30].

Parental stress might affect children’s social development through its effect on parenting behaviours. Parenting stress has been found to predict dysfunctional parenting practices, including negative parenting styles [27] and poorer parenting behaviours [31–33], which in turn are associated with problematic child behaviours [26, 34]. Parents with higher stress levels display less responsiveness in their parent–child interactions and more authoritarian parenting styles (e.g. overly strict and controlling), and show an increased risk of child maltreatment (e.g. harsh verbal and physical disciplining practices) [34–37]. These negative parenting styles are associated with poor behavioural, socio-emotional, and cognitive outcomes among children, and can negatively influence coping skills among children whose parents have high levels of parenting stress [23]. Additionally, they can lead to more emotional, behavioural, cognitive, and physical problems throughout the child’s development [34].

Given the above theory, we propose that mother’s SOC may influence children’s social development indirectly through its influence on parenting practices. However, despite SOC’s important role in parenting stress and the fact that such stress has been demonstrated to negatively influence child development, there has been very little research on the mechanisms of the relationship between mother’s SOC and children’s development. Several reports have established relationships between parental SOC and children’s social development [21, 38], but the number is paltry in absolute terms. Particularly, there is little research focusing on the relationships between parental SOC and children’s development in preschool children.

Parental SOC not only may affect children’s social development through parenting attitudes and behaviours, but also may directly affect it through demonstration of better coping methods to children. In other words, children may benefit from perceiving the life orientation of a parent with high SOC, as such an orientation may be connected to flexible and successful coping. This may also lower the risk of negative outcomes for children, such as social maladjustment. However, as noted before, there is comparatively little research on the influence of parents’ SOC on child development among preschoolers, which makes the above mere speculation.

The development of social skills in early childhood is an important area of research in the field of child development, as such skills are essential for social competence [39–41]. Social competence is defined as an individual’s ability to function in relation to other people, particularly with respect to getting along with others and forming close relationships [42]. It is also viewed as the ability to understand others in the context of social interactions and engage in smooth communication with others [43]. Social competence has been shown to be an important protective factor for children, as it is a buffer against stress and thereby helps to prevent serious emotional and behavioural problems later in life [44]. Deficits in social skills (e.g. cooperation, self-control, and assertion) in early childhood are relatively stable over time, and appear to relate to problems such as externalizing and internalizing disorders and poor academic performance, which are in turn precursors to more severe problems in the future [45–49].

The development of social skills is determined by complex interactions between the individual, home and school environments, peer relationships, and sociocultural background [50]. Primarily, however, children develop their social and emotional competence through interactions with others. Indeed, such skills are likely heavily dependent in early childhood on the family context, including parental involvement [24, 51]. Although mother’s SOC and parenting style may affect children’s social competence development, few studies have comprehensively confirmed this relationship. Thus, it is important to examine the associations among mother’s SOC, childrearing style, and child social skills development in a comprehensive model.

### Current study

We aimed to clarify the relationship between mother’s SOC and children’s social skills development among preschoolers, particularly whether this relationship is mediated by childrearing style. We hypothesized the following pathways: (1) an indirect pathway between mother’s SOC and child’s social skills development through mother’s childrearing style; and (2) a direct pathway between mother’s SOC and child’s social skills development after controlling for childrearing style.

## Methods

### Participants

In 2013, self-report questionnaires were administered to mothers of preschool children (n = 1845) aged 4–5 years in 21 nursery schools and 10 kindergartens in Kyoto, a highly urbanized metropolis in Japan. Of those 1845 mothers, 1362 completed the questionnaires.

In the present paper, to accurately clarify the associations between mother’s SOC, mother’s childrearing style, child’s social skills (i.e. cooperation, self-control, and assertion), the following were excluded from the analysis: (1) children diagnosed with developmental problems (these children had already been diagnosed at medical institutions before this study started), and (2) children whose mothers did not return completed questionnaires. For inclusion in this study, mothers did not have to be the target child’s biological parent; however, they did need to reside with the child. Of the 1362 children’s mothers who completed questionnaires, we excluded 21 because the children had a diagnosed developmental disorder. Thus, 1341 met the inclusion criteria. The children’s data were analysed in this study.

#### Ethics statement

Informed consent was obtained from all mothers and teachers prior to the start of this research. They were informed of the purpose and procedures of the study, and were made aware that they were not obliged to participate. Ethical approval was obtained from Kyoto University’s ethics committee in Kyoto, Japan (E1701).

### Measures

The demographic information collected included mother’s and child’s age and sex, family structure, and perceived family economy.

#### Predictor: mother’s sense of coherence

The short version of the sense of coherence scale (SOC-13) [1, 52] was used. This is a short form of the original 29-item scale (SOC-29) and has demonstrated reliability and validity [53]. The scale has been validated for the Japanese population [54, 55]. The scale comprises 13 items measuring the domains of comprehensibility (5 items, e.g. ‘Has it happened in the past that you were surprised by the behaviour of people whom you thought you knew well?’), manageability (4 items, e.g. ‘Has it happened that people whom you counted on disappointed you?’), and meaningfulness (4 items, e.g. ‘Do you have the feeling that you really don’t care about what is going on around you?’). Items are rated on a 7-point scale ranging from 1 to 7. The SOC score is obtained by summing the 13 items; SOC is regarded as a unitary construct, and higher scores indicate a stronger SOC. The measure has adequate internal consistency and construct validity [53, 55]. In this study, the internal consistency was .82. In addition, the quality of the factor analysis models was assessed using the Kaiser-Meyer-Olkin (KMO) test and Bartlett ´s test for sphericity. The KMO test measures the degree of multicollinearity between the included items and varies between 0 and 1; the recommended minimum is .50 [56]. In this study, the KMO value was .86. This was an acceptable KMO value. Bartlett’s test is a measure of the probability that the initial correlation matrix is an identity matrix and should be <.05. In this study, Bartlett’s Test was significant, indicating that the matrix does not resemble an identity matrix, further supporting the existence of factors within the data. In addition, homoscedasticity was inspected through Levene’s test; the test was greater than .05. Levene’s test was not significant, indicating results that showed homoscedasticity. The total score was converted to a *z*-score for the analysis.

#### Mediator: mother’s childrearing style

The Index of Child Care Environment (ICCE) is a 13-item measure of childrearing style [57]. The scale comprises 13 items that measure the level of human stimulation, social stimulation, avoidance of restriction, and social support provided in a child’s environment (e.g. ‘How often do you play with your child?’ ‘How many times have you hit or kicked your child?’). The ICCE was originally created and developed in Japan. This scale is based on the home observation for measurement of the environment, which is used to evaluate the quality and quantity of stimulation and support available to children in their home environment [58]. Each item is assessed using a multiple-choice format, and the answer is given a binary score according to the manual (1 = good, 0 = not good or not sure); the overall score is calculated by summing all item scores. A higher score indicates better childrearing. The measure has adequate internal consistency and construct validity [57]. In this study, the internal consistency was .71. In addition, in this study, the KMO value was .79, Bartlett’s test was significant, and Levene’s test was not significant; these values indicated that assumptions of sphericity and homoscedasticity were met. The total score was converted to a *z*-score.

#### Criterion variable: child’s social skills

The social skills scale (SSS) is a 24-item measure of children’s social competence in terms of ‘cooperation’ (8 items, e.g. ‘Helps friends when asked’), ‘self-control’ (8 items, e.g. ‘Postpones gratification when requested’), and ‘assertiveness’ (8 items, e.g. ‘Expresses appropriate greetings to others’) [43, 59], which are all factors affecting social adaptation in later life [60]. In this study, children’s teachers were recruited to evaluate their social skills using this scale. The three subscales positively correlate with the scores of the child development scale [43, 59], which is based on the Social Skills Rating System [60]. Items are rated on a 3-point scale ranging from 0 to 2; the item scores are summed for each subscale to arrive at total scores for assertiveness, self-control, and cooperation. Higher scores indicate better social skills. The measure has adequate internal consistency and construct validity [43, 59]. In this study, internal consistency ranged from α = .83–.93. In addition, in this study, the KMO values were excellent in their range (cooperation; .93, self-control; .93, assertion; .90), the Bartlett’s Tests were significant, and the Levene’s tests were not significant; these values indicated that assumptions of sphericity and homoscedasticity were met. Each SSS total score was converted to a *z*-score.

### Procedure

There were 260 nursery schools and 122 kindergartens in Kyoto city, Japan. We asked the facilities and conducted our survey at facilities where permission was obtained. To recruit families, self-reported questionnaires were distributed to all parents of preschool children (n = 1845) aged 4–5 years in the 21 nursery schools (12 private nursery schools and 9 public nursery schools) and 10 kindergartens (10 private kindergartens). The principals of each participating facility gave permission for us to meet with the parents. The participants received an information sheet and questionnaires with their child’s ID number. Participants provided written informed consent and agreed to participate. The parents completed the questionnaires at a single time point and returned these to participating facilities in sealed envelopes to prevent the teachers from seeing the questionnaires. Then, the child’s teacher checked the ID number of each received questionnaire, and the teachers evaluated the children’s social skills using the SSS.

### Data analyses

**Fig. 1:**
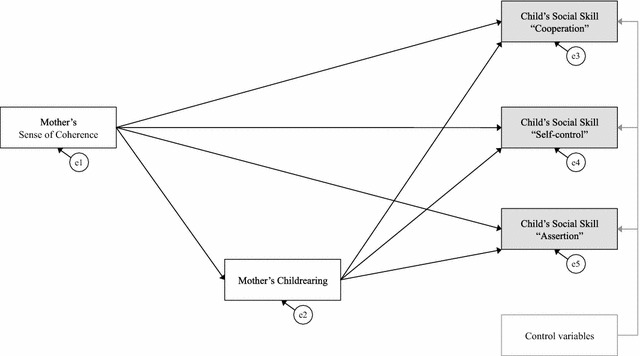
Hypothesized model. This model includes the hypothesized pathways between mother’s sense of coherence, mother’s childrearing, and children’s social skills (cooperation, self-control, and assertion). All observed variables are in *boxes*, and error terms (e1–e5) are in *ellipses*

Correlational analysis was performed to measure associations between mother’s SOC, mother’s childrearing style, child’s social skills (i.e. cooperation, self-control, and assertion), and demographic characteristics (i.e. child’s sex, child’s age, mother’s age, presence of father, presence of siblings, attending kindergarten, and perceived family socioeconomic status). Path analyses were then conducted to estimate direct and indirect paths between mother’s SOC, mother’s childrearing style, and child’s social skills. In the models, mother’s SOC was specified as a predictor of (a) mother’s childrearing style and (b) child’s social skills. Prior to estimating the full model (Fig. 1), a partial model that did not include mother’s childrearing style was estimated. All observed variables are enclosed in boxes and unobserved variables in ellipses. The unobserved variables are error terms. Error terms are associated with all endogenous variables and represent measurement error along with effects of variables not measured in the study.

To assess fit, we used the comparative fit index (CFI) [61], incremental fit index (IFI) [62], and the root mean square error of approximation (RMSEA) [63]. Good fit is reflected by CFI and IFI values above .90 [61, 62] and RMSEA values of .08 or less [64]. All statistical analyses were conducted using SPSS version 22.0 and AMOS version 23.0.

## Results

### Descriptive statistics

In this study, 1341 mothers and children were analysed. For children, there were 649 girls (48.4%); 680 (50.7%) were 4-year-olds, while 661 (49.3%) were 5-year-olds. The mothers’ ages ranged from 22 to 50 (M = 36.82, *SD* = 4.75). In terms of presence of father, 1221 (91.1%) children resided with father. In terms of presence of siblings, 1041 (77.6%) children had siblings. In terms of children’s attendance, 717 (53.5%) children attended kindergartens, while 624 (46.5%) children attended nursery schools. In terms of perceived family socioeconomic status, 134 mothers (10.0%) classified themselves as very poor, 164 (12.2%) as poor, 860 (64.1%) as fair, 156 (11.6%) as good, and 27 (2.0%) as very good.

Descriptive statistics for all study variables are presented in Table 1. Correlation analysis was used to determine relations between the main variables (i.e. mother’s SOC, mother’s childrearing style, child’s social skills) and the demographic variables (i.e. child’s sex, child’s age, mother’s age, presence of father, presence of siblings, attending kindergarten, and perceived family socioeconomic status) to identify potential control variables in Table 2. All of the main variables were significantly correlated. Furthermore, significant correlations were also found between the demographic variables except mother’s age, and the dimensions of social skills (i.e. cooperation, self-control, and assertion). Specifically, child’s sex was significantly related to cooperation (*r* = .19, *p* < .001), self-control (*r* = .27, *p* < .001), and assertion (*r* = .11, *p* < .001), as was child’s age (cooperation, *r* = .25, *p* < .001; self-control, *r* = .22, *p* < .001; assertion, *r* = .14, *p* < .001), presence of father (cooperation, *r* = .09, *p* < .01; self-control, *r* = .09, *p* < .01; assertion, *r* = .06, *p* < .05), presence of siblings (cooperation, *r* = .09, *p* < .01; self-control, *r* = .05, *p* < .05; assertion, *r* = .05, *p* < .05), attending kindergarten (cooperation, *r* = .10, *p* < .001; self-control, *r* = .07, *p* < .05; assertion, *r* = .05, *p* < .05) and family socioeconomic status (cooperation, *r* = .06, *p* < .05; self-control, *r* = .07, *p* < .05; assertion, *r* = .10, *p* < .01). Therefore, these demographic variables were entered into the predictive models as control variables.

**Table 1:** Descriptive statistics for the study variables (N = 1341)

Description	Range	Mean	SD	Cronbach’s α
Mother’s sense of coherence				
Sense of coherence	13–91	59.32	11.95	0.82
Mother’s childrearing				
Index of child care environment	0–13	11.43	1.16	0.71
Child’s social skills				
Social skills scale				
Cooperation	0–16	10.98	4.19	0.93
Self-control	0–16	14.18	2.54	0.90
Assertion	0–16	14.09	2.32	0.83

**Table 2:** Correlations between mother’s sense of coherence, mother’s childrearing, child’s social skills, and demographic characteristics

Variable	1	2	3	4	5	6	7	8	9	10	11	12
Mother’s sense of coherence												
1. Sense of coherence	–											
Mother’s childrearing												
2. Index of Child Care Environment	.25***	–										
Child social skills												
3. Cooperation	.12***	.10***	–									
4. Self-control	.10***	.10***	.57***	–								
5. Assertion	.10***	.10***	.66***	.57***	–							
Demographic characteristics												
6. Child’s sex	−.02	.02	.19***	.27***	.11***	–						
7. Child’s age	.05	.01	.25***	.22***	.14***	.01	–					
8. Mother’s age	.05	.01	−.01	.06	−.01	.01	.10**	–				
9. Presence of father	.09**	.02	.09**	.09**	.06*	.01	−.02	.08**	–			
10. Presence of siblings	.10**	−.05	.09**	.05*	.05*	.05	.00	.05***	.13***	–		
11. Attending kindergarten	.06*	.03	.10***	.07*	.05*	−.03	−.04	.06*	.17***	.07*	–	
12. Perceived family socioeconomic status	.22***	.08**	.06*	.07*	.10**	−.01	−.05	.07*	.13***	−.01	.07**	–

Variables were coded as follows: *1* sense of coherence, *2* Index of Child Care Environment, *3* cooperation, *4* self-control, *5* assertion, *6* child’s sex, *7* child’s age, *8* mother’s age, *9* presence of father, *10* presence of siblings, *11* attending kindergarten, *12* perceived family socioeconomic status

* *p* < .05

** *p* < .01

*** *p* < .001

### Hypothesized paths

To test the mediating effect, we tested three models (Table 3). In Model 1, the standardized direct effect of mother’s SOC on child’s social skills, without controlling for mother’s childrearing style, was statistically significant (cooperation, *β* = .34, *p* < .05; self-control, *β* = .32, *p* < .01; assertion, *β* = .19, *p* < .05). In Model 2, the study indicated the effect of mother’s SOC on child’s social skills in the full mediation model was both directly and indirectly statistically significant. Therefore, mother’s childrearing style appears to be a mediator in the relationship between mother’s SOC and child’s social skills.

**Table 3:** Coefficients for path analyses

Model/construct			B	SE	*β*	*p*
Model 1; direct model						
Mother’s sense of coherence	→	Child’s social skill “Cooperation”	2.40	.14	.34	*
Mother’s sense of coherence	→	Child’s social skill “Self-control”	2.90	.11	.32	**
Mother’s sense of coherence	→	Child’s social skill “Assertion”	2.04	.09	.19	*
Model 2; mediation model						
Mother’s sense of coherence	→	Mother’s childrearing	6.88	.03	.20	***
Mother’s sense of coherence	→	Child’s social skill “Cooperation”	1.47	.15	.22	
Mother’s sense of coherence	→	Child’s social skill “Self-control”	1.85	.11	.21	**
Mother’s sense of coherence	→	Child’s social skill “Assertion”	.73	.10	.07	
Mother’s childrearing	→	Child’s social skill “Cooperation”	1.67	.16	.26	**
Mother’s childrearing	→	Child’s social skill “Self-control”	1.75	.12	.21	*
Mother’s childrearing	→	Child’s social skill “Assertion”	1.98	.10	.20	**

The path analyses were controlled for child sex and age, presence of father, presence of siblings, attending kindergarten, and perceived family economy

* *p* < .05

** *p* < .01

*** *p* < .001

**Fig. 2:**
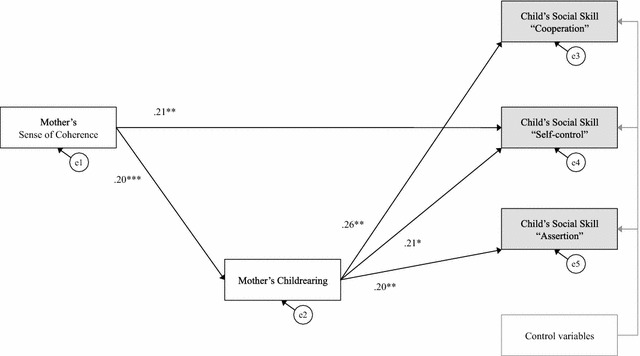
Statistically significant paths. This model includes paths that were statistically significant in the hypothesized model. Path analyses controlled for child sex and age, presence of father, presence of siblings, attending kindergarten, and perceived family socioeconomic status. All observed variables are in *boxes*, and error terms (e1–e5) are in *ellipses*. Model fit statistics: χ^2^ (22) = 148.84, CFI = .94; IFI = .94; RMSEA = .06. *p < .05; **p < .01; ***p < .001

In Model 2, several statistically significant direct paths were found between the predictors and criterion variables (Fig. 2). First, mother’s SOC was found to be a significant predictor of mother’s childrearing style (*β* = .20, *p* < .001) and child’s self-control (*β* = .21, *p* < .01). Mother’s childrearing style was also found to be a significant predictor of child’s cooperation (*β* = .26, *p* < .01), self-control (*β* = .21, *p* < .05), and assertiveness (*β* = .20, *p* < .01). Therefore, mother’s SOC was found to indirectly relate to child’s social skills through mother’s childrearing style. Additionally, according to the fit indices, the full model fit the data well [χ^2^ (22) = 148.84, CFI = .94; IFI = .94; RMSEA = .06]. Figure 2 displays the final model and the standardized path coefficients.

## Discussion

We examined the correlations between mother’s SOC, mother’s childrearing style, and child’s social skills among preschool children, and tested whether mother’s childrearing was a mediator in the relationship between the other two variables. Our hypothesized model was confirmed, in that mother’s SOC was a significant predictor of social skills through mother’s childrearing style. In addition, notably, mother’s SOC was a significant predictor of social skills directly, even after adjusting for mother’s parenting.

### Indirect path between variables

Mother’s SOC was positively linked with cooperation, self-control, and assertiveness indirectly through mother’s childrearing style. This result is consistent with previous research findings, as related in the following sections.

#### Association between mother’s SOC and childrearing style

Mother’s SOC was positively linked with mother’s childrearing style, which has been indirectly supported in previous findings. As noted previously, individuals with a strong SOC were more likely to perceive their lives as less stressful [19], and SOC appears to be inversely related to parenting stress [21]. Parents with strong SOC are likely to select more active coping strategies and report more positive feelings toward positive parenting, such as emotional expressiveness, responsiveness, and support [65, 66]. Positive parenting has been shown to enhance empathy and children’s social functioning [67–70]. In contrast, parents with poor SOC are likely to be vulnerable to parenting stress [21]. Parenting stress influences parenting behaviours that are not developmentally appropriate, inconsistent discipline (i.e. alternating between too lax and too harsh), and a lack of warmth and responsiveness in parent–child interactions [31–33, 35, 71, 72]. Furthermore, parents experiencing high levels of stress are typically less responsive and affectionate with their children and more likely to use power-assertive discipline strategies and hold hostile parental attitudes, as compared with parents who can effectively cope with their stress [73]. Parenting stress is also presumed to interfere with parenting practices that help regulate children’s behaviour and emotions [74]. Therefore, parents with a stronger SOC who can effectively cope with their stress, are likely to have more positive parenting styles, whereas parents with a poorer SOC who are more vulnerable to stress, are more likely to have more negative parenting styles.

Furthermore, individuals with a stronger SOC use personal and emotional intelligence in proper ways to smoothly handle multiple demands and quickly adapt to their social environments [75]. Parents with higher SOC would make better choices, more adeptly manage their lives, and face fewer problems in life events, and have less time to solve them, than those with poorer SOC [76, 77]. Thus, higher SOC is likely to mean less time devoted to dealing with such problems and their consequences, and consequently, more time and availability to care for their children. On the other hand, worse SOC may mean less time and availability to care for children, or at least worse care. For the above reasons, mother’s SOC may be positively linked with mother’s childrearing style.

#### Association between mother’s childrearing style and child’s social skills

We also found an association between mother’s childrearing style and specific social skills—cooperation, self-control, and assertiveness—among children. This result is consistent with previous findings that parenting is an important contributor to children’s social development [70]. For example, greater parental warmth and sensitivity predicts greater emotional sensitivity, perspective taking skills (i.e. awareness and understanding of other people’s situations), and prosocial behaviours among children [78–80]. Furthermore, parents who use consistent discipline such as firm rules and structure while encouraging development of mastery and independence appear to have more socially competent children [74]. Children whose parents are supportive, emotionally available, and teach their children effective emotion regulation strategies and coping skills are more likely to be socially competent and less prone to experience negative emotions/behaviours with peers [81–83].

In contrast, the use of power-assertive discipline strategies and hostile parental emotions appear to be negatively associated with empathic and prosocial development [78], while stricter control predicted somewhat lower levels of positive social behaviour [79]. For instance, parents who are overly strict and controlling might place undue demands on children, which might cause children to develop negative affect (e.g. anger) and engage in more self-focused thoughts and actions [79]. Furthermore, parental control mixed with harsh verbal and physical disciplining practices can lead to aggressive and antisocial behaviours [79] or other problem behaviours among children [84].

Thus, a warm and supportive parenting style is viewed as an important resource associated with positive developmental outcomes, whereas overly controlling parenting is associated with negative outcomes. Therefore, mother’s SOC (that is, the ability to manage stress), through its effect on parenting style, can affect a child’s social development.

### Direct path between mother’s SOC and child’s social skills

Interestingly, mother’s SOC was directly positively linked with the development of self-control. This result accords with the results of previous studies, indicating that parents’ personal ability is associated with a child’s social development [38, 78, 85]. A possible direct mechanism of the effect of mother’s SOC on children’s development is modelling. Social learning theory suggests that children’s social development is influenced by modelling of behaviours and attitudes of significant others in their lives [86, 87]. Thus, child social development is likely to be positively related to parents’ personal ability via the effects of modelling [88].

Consistent with the theory, the direct relationship between mother’s SOC and children’s social skills might be due to the effects of modelling. Several studies on self-regulation of emotions suggest that parents provide significant models by which children learn to express emotions and later learn to control emotional expressivity [89, 90]. SOC helps individuals to express and control emotions effectively, by facilitating various resources within the individuals to cope with life events. Parents are faced with multiple stressors and they may differ in their responses to these stressors—some parents with higher SOC are able to deal with challenges more effectively while others with lower SOC display emotional intensity or become more inappropriately reactive. For instance, children whose parents display a wide range of positive and negative emotions in appropriate social contexts, are more likely to be able to learn how to express emotions that are appropriate to display in particular situations [91]. As a result, children are more likely to be able to control their emotions effectively and efficiently. In contrast, children whose parents display higher levels of anger or personal distress, are less likely to be able to observe and learn appropriate ways to regulate and express their negative emotions [92, 93]. Therefore, parents with higher SOC might model positive and socially appropriate emotional responses to frustrating situations and provide adaptive emotional coaching. In contrast, parents with lower SOC might model undercontrolled, angry emotions in frustrating situations and respond in a punitive fashion to their children’s expression of negative emotions. Consequently, mothers’ SOC levels were directly positively linked with the development of self-control, by demonstrating and teaching coping methods to their children.

## Limitations and future directions

The findings should be interpreted in light of several limitations. First, this was a cross-sectional study. The cross-sectional design poses several restrictions that make it difficult to assume causality among the factors. Prior studies have found that children’s developmental characteristics influence mother’s SOC as well as the influence of mother’s personal ability on children’s developmental outcomes [94–96]. Children’s mental health functioning and mother’s SOC are likely to influence each other. Thus, longitudinal research is needed in order to examine the effects of mother’s SOC on the later development of preschool children.

Second, the majority of the data (i.e. mother’s SOC, child-rearing style, and demographic information) were obtained only from mothers; therefore, there is a risk of a reporting bias. Specifically, single respondents’ views toward child-rearing style and demographic information such as perceived family socioeconomic status, may be skewed either more positively or negatively. Therefore, in future studies, more dissimilar informants’ reports, including those from fathers, in addition to mothers, will be needed to evaluate more exactly how family factors affect children’s mental health functioning.

Third, in the current study, we evaluated family socioeconomic status only using perceived (i.e. self-reported) family socioeconomic status. Numerous studies have consistently found childhood socioeconomic status has been associated with children’s developmental outcomes [97–99]. The previous studies have consistently focused on three quantitative indicators to provide reasonably good coverage of the domains of interest: income, education, and occupational status. Therefore, in future studies, more proper scales, including family income, parent’s education, and occupational status, will be needed to evaluate more exactly how socioeconomic status affects child mental health functioning.

Fourth, there are likely to be several other factors that were not accounted for in our model. Although we found the hypothesized effects of mother’s SOC on child mental health functioning, we did not consider certain other mother’s social abilities in our model. According to social learning theory, parents’ personal abilities influence children’s social functioning by modelling of behaviours [86, 87]. Mothers’ social skills might affect their children’s social skills through both modelling and interaction with the children. In addition, although children’s social competence is influenced by their modelling of behaviours and attitudes of significant others in their lives, we did not consider social characteristics of fathers and teachers, such as their SOC, in our model. Children spend much time together not only with mothers but also their fathers and teachers; hence, their fathers’ and teachers’ personal abilities might significantly influence children’s developmental outcomes.

Furthermore, although we did not consider genetic factors in our model; it is important to realize children’s social competence may be influenced by genetic risks as well as their environmental factors. Considerable evidence supports the conclusion that children’s mental health functioning is moderately heritable [100, 101]. The extent to which children’s mental health functioning is affected by environmental factors depends on genetic characteristics [102, 103]. Consequently, there are likely to be other factors that need to be included in this model. Future studies should investigate this possibility further by including more factors related to children’s mental health functioning.

Finally, these findings may not be generalizable to all families, because the sample was drawn from a limited geographical area in an urban metropolis in Japan. The reproducibility of the current results should be confirmed using data from other regions in a variety of settings. In summary, future research on these topics would benefit from longitudinal designs and samples with greater demographic and clinical diversity.

## Conclusions

This study examined the interrelations between mother’s SOC, mother’s childrearing style, and child’s social skills in early childhood. We found significant direct paths from mother’s SOC to mother’s childrearing style and child’s social skills. These findings advance our understanding of how mother’s SOC and parenting affect children’s development according to a family systems perspective. Lacking social skills in early childhood puts children at risk for social maladjustment [46, 47, 104]. Therefore, focusing on support and education to maintain and improve mothers’ SOC, especially mothers with lower SOC, may be an efficient and effective strategy for improving children’s social adjustment.

## Abbreviations

SOC: The Sense of Coherence Scale
ICCE: The Index of Child Care Environment
SSS: The Social Skills Scale
